# Associations of Polymorphisms in* HRH2*,* HRH3*,* DAO,* and* HNMT* Genes with Risk of Chronic Heart Failure

**DOI:** 10.1155/2016/1208476

**Published:** 2016-02-17

**Authors:** Gong-Hao He, Wen-Ke Cai, Jia-Bin Zhang, Chao-Yu Ma, Feng Yan, Jun Lu, Gui-Li Xu

**Affiliations:** ^1^Department of Pharmacy, Kunming General Hospital of Chengdu Military Region, Kunming 650032, China; ^2^Department of Cardio-Thoracic Surgery, Kunming General Hospital of Chengdu Military Region, Kunming 650032, China; ^3^Surgery Center, 302 Hospital, Beijing 100039, China; ^4^Information Department, Kunming General Hospital of Chengdu Military Region, Kunming 650032, China; ^5^Department of Pharmacy, The First Affiliated Hospital, College of Medicine, Xi'an Jiaotong University, Xi'an 710061, China

## Abstract

The pathophysiological functions of cardiac histamine level and related histamine receptors during the development of chronic heart failure (CHF) were intensively investigated previously. However, the relevance of polymorphisms in histamine-related genes, such as* HRH2*,* HRH3*,* DAO,* and* HNMT*, with CHF remains largely neglected. This study herein aims to analyze the clinical associations of polymorphisms in those genes with CHF risk. A total of 333 unrelated Chinese Han CHF patients and 354 ethnicity-matched healthy controls were recruited and 11 single nucleotide polymorphisms (SNPs) were genotyped. We found that the* HRH3* rs3787429 polymorphism was associated with CHF risk (*p* < 0.001). The T allele of rs3787429 exhibited protective effect against CHF under the dominant (ORs = 0.455; 95% CIs = 0.322–0.642) and additive models (ORs = 0.662; 95% CIs = 0.523–0.838), while, for SNPs in* HRH2*,* DAO,* and* HNMT*, no significant associations were observed in the present study. These findings for the first time screen out one SNP (rs3787429) of* HRH3* gene that was significantly associated with CHF in Chinese Han population, which may be a novel biomarker for personal prevention and treatment of CHF and provides novel highlights for investigating the contribution of this disease.

## 1. Introduction

Chronic heart failure (CHF) is a common clinical syndrome that can result from and is the end-stage of any structural or functional cardiac disorders [1]. Although it is well acknowledged that CHF is a multifactorial process, factors involved in the initiation and progression of CHF are rather complicated and yet to be identified. Recently, investigations from different laboratories continuously suggested that cardiac histamine and its related receptor subtypes (e.g., H_2_, and H_3_ receptors) played considerable roles during the development of CHF [2–5], indicating that histamine and its related molecules are important factors associated with this disease. Based on these findings, our previous investigation further explored the genetic association between CHF and certain polymorphisms in* histidine decarboxylase* (*HDC*) gene, which encodes the only rate-limiting enzyme in histamine synthesis, and found that the* HDC* rs17740607 polymorphism decreased the enzyme activity of HDC, lowered the plasma histamine levels, and was significantly associated with CHF risk [6], which further strengthened the evidence of histamine involvement in CHF.

Nevertheless, besides HDC, endogenous histamine levels may be affected by several other factors such as the two main catabolic enzymes: diamine oxidase (DAO) and histamine N-methyltransferase (HNMT). Moreover, the function of cardiac histamine varies with different histamine receptor subtypes involved. On one hand, it was reported that histamine H_2_ receptors (HRH2) expressed in cardiac myocytes are able to induce positive inotropic and chronotropic effects when activated [3, 7, 8], which may increase the myocardial oxygen consumption and aggravate CHF. On the other hand, cardiac histamine was reported to exert modulating effects on cardiac sympathetic activity [9] and local rennin-angiotensin system [10] and hence ameliorate myocardial remodeling via histamine H_3_ receptors (HRH3). Therefore, these histamine-related proteins (HRH2, HRH3, DAO, and HNMT) may be new important diagnostic or therapeutic targets of CHF. With respect to the notion that genetic factors contribute greatly to the risk and development of CHF [11], polymorphisms in* HRH2*,* HRH3*,* DAO,* and* HNMT* genes are likely to be also associated with CHF risk, which, however, is still uninvestigated despite the fact that the roles of some of these proteins in CHF were intensively investigated.

Based on this background, this study aims to further clarify the relationships between certain tag-single nucleotide polymorphisms (SNPs) or previously reported positive SNPs of* HRH2*,* HRH3*,* DAO,* and* HNMT* genes and CHF risk using case-control method among Chinese Han population hoping to provide novel genetic risk markers for personalized prevention and treatment of CHF, which would eventually inform our understanding of the disease.

## 2. Materials and Methods

### 2.1. Study Population

A total of 333 randomly selected patients with CHF who were admitted to Kunming General Hospital of Chengdu Military Region between 2012 and 2015 and 354 unrelated, sex-, age-, and ethnicity-matched healthy individuals who had no known cardiovascular-related diseases or hereditary disorders and who were not taking any cardiovascular-related medications were included. The primary inclusion and exclusion criteria for CHF patients are in accordance with our previous report [6] as well as the Framingham criteria [12]. Briefly, the inclusion criteria were diagnosis of CHF with abnormal left ventricular function by echocardiography. The exclusion criteria were age < 18 years and the presence of severe hepatic or renal insufficiency, tumors or malignant disease, acute attack of CHF, or severe acute infection. Extensive clinical data were collected at enrollment by clinical assessment and echocardiography to define the etiology of CHF, which was classified as ischemic or nonischemic subgroups. Ischemic CHF was defined by at least a 50% narrowing on coronary angiography, a positive stress test, history of an acute coronary syndrome, or previous coronary revascularization. Cases free of these criteria were classified as nonischemic. Cases that could not be clearly classified were excluded from the present study. For control participants, the inclusion criteria were no history of heart failure, normal ventricular function on cardiac imaging, and no evidence of coronary artery disease as determined either by a negative treadmill exercise test or by maximal coronary stenoses of ≤20% on coronary angiography. Exclusion criteria for the controls were personal and family history of cardiovascular disease.

The subjects were considered to have hypertension as a systolic blood pressure (SBP) of 140 mmHg or a diastolic blood pressure (DBP) of 90 mmHg or those who were receiving antihypertensive therapy at the time of the examination. Dyslipidemia was defined according to Chinese Guidelines on Prevention and Treatment of Dyslipidemia in Adults [13]. Diabetes was defined in agreement with the American Diabetes Association [14].

The study protocols were drawn up in compliance with the principles of the Helsinki Accord and were reviewed and approved by the Ethical Committee of Kunming General Hospital of Chengdu Military Region. Statement of informed consent was obtained from all participants after a full explanation of the procedure.

### 2.2. Genotyping Assay

Genotyping experiments were performed as previously described [6, 15, 16]. Briefly, the blood samples were collected into tubes containing ethylenediaminetetraacetic acid and stored at −80°C until analysis. Standard phenol–chloroform extraction method was used to extract genomic DNA from whole blood. DNA concentration was measured by spectrometry (DU530 UV/VIS spectrophotometer, Beckman Instruments, Fullerton, CA, USA). Eleven candidate SNPs in* HRH2*,* HRH3*,* DAO,* and* HNMT* genes were selected in reference with HapMap data for Chinese Han population (http://www.hapmap.org/) and genotyped by Sequenom MassARRAY RS1000 according to the standard protocol recommended by the manufacturer. Sequenom MassARRAY Assay Design 3.0 Software was used to design Multiplexed SNP MassEXTEND assay. The primers used for the 11 SNPs are listed in Table 1. Sequenom Typer 4.0 Software was used to perform data management and analyses.

### 2.3. Statistical Analysis

Statistical analyses were performed with SPSS 18.0 for Windows (PASW Statistics, SPSS Inc., Chicago, IL). A two-sided *P* value < 0.05 was considered statistical significant for all tests. Bonferroni's corrections were used for multiple comparisons. Each SNP frequency in control subjects was tested for departure from Hardy-Weinberg Equilibrium (HWE). Student's *t*-test, analysis of variance (ANOVA), chi-square (Pearson's *χ* ^2^) test or Fisher's exact test, and multivariate logistic regression analysis adjusted for age, gender, body mass index (BMI), and traditional cardiovascular risk factors (hypertension, dyslipidemia, diabetes, and smoking habit) under different genetic models were used where necessary according to our previous work [6]. Odds ratios (ORs) with 95% confidential intervals (CIs) were used to assess the associations between genotypes and CHF risk or clinical variables.

## 3. Results

### 3.1. Population Characteristics

The distributions of selected demographic and clinical characteristics of both the case and control groups are given in Table 2. Cases and controls were matched for age, gender composition, and BMI (*P* > 0.05). However, the prevalence of all traditional cardiovascular risk factors was observed to be significantly higher in CHF patients than that in the control group (*P* < 0.05). Based on selection criteria, the health controls had normal left ventricular ejection fraction (LVEF) whereas the CHF cases had severe systolic heart failure, with an average LVEF of 33.3 ± 7.0%. In addition, the prevalence of ischemic and nonischemic etiologies was relatively close.

### 3.2. Distributions of Genotype and Allele between CHF Patients and Health Controls

Table 3 shows the distributions of genotype and allele frequencies of the 11 selected SNPs in CHF patients and health controls. For each SNP distribution in controls, HWE test was performed, which revealed that none of the genotype distributions differed significantly from those expected under HWE (*P* > 0.05, Table 3). As for the association analysis, we found a significant correlation between the rs3787429 polymorphism (one of the only 2 tag-SNPs in* HRH3* gene) and CHF risk according to both genotype and allele (*P* < 0.001, resp.) distributions at Bonferroni-corrected *P* level of 0.0045 (0.05/11 SNPs), which still remained significant after further adjustment for age, gender, BMI, and a series of traditional cardiovascular risk factors. The T allele of rs3787429 was more frequent in control group than in CHF patients and exhibited protective effect against CHF (adjusted OR, 0.608; 95% CI, 0.470–0.786; *P* < 0.001; Table 3). These data further strengthen the independent role of the rs3787429 polymorphism in modulating CHF susceptibility per se, despite the presence or absence of traditional cardiovascular risk factors. However, for the rest of the SNPs, no significant associations with CHF risk were observed before or after adjustment in the present population.

In the following exploration, we also performed multivariate analysis on* HRH3* rs3787429 polymorphism under three genetic models of inheritance (dominant, recessive, and additive model). After adjustment, the rs3787429 polymorphism was still significantly and independently associated with the predisposition to CHF under a dominant and additive but not recessive model in the present populations and consistently exhibited protective effect against CHF according to the values of ORs and 95% CIs (Table 4).

### 3.3. Subgroups and Intermediate Phenotypes

To further explore the association between rs3787429 polymorphism and CHF, we explored the genotype distributions of rs3787429 in relation to different CHF clinical subsets (Table 5). In the present CHF patients, we found that neither the genotype nor the allele distribution of the rs3787429 differed significantly with functional New York Heart Association (NYHA) class (*P* = 0.282 and 0.247, resp.). Furthermore, we did not observe any significant difference regarding the LVEF between the 3 genotypes of rs3787429. However, the distributions of the rs3787429 genotype and allele in all of the CHF subsets (i.e., hypertensives, dyslipidemias, diabetics, and smokers) were significantly different with those in controls. Moreover, we also used all available data to explore whether the strength of association for our findings varied with the etiology of CHF (i.e., ischemic and nonischemic). As shown in Table 5, the genotype and allele associations with rs3787429 were still noteworthy in both ischemic and nonischemic subtypes of the present population.

## 4. Discussion

This study genotyped 11 SNPs from 4 histamine-related genes in a CHF case-control population and for the first time screened out that the* HRH3* rs3787429 polymorphism is associated with CHF risk. The T allele of rs3787429 exhibited protective effect against CHF. These findings provide novel genetic data supporting and strengthening the previous observation that cardiac HRH3 is one of the key elements involved in modulating the progress of various cardiac dysfunctions including CHF [4, 17–19].

The SNP selection in the present study is on the basis of HapMap data regarding Chinese Han population (http://www.hapmap.org/) and previous documents as well [20]. For characters of the control group, the minor allele frequency (MAF) of most selected SNPs in control group was roughly close to the HapMap data except for the* DAO* rs1049742, whose MAF was extremely low (only 1 heterozygous variant was observed) in the present population. Nevertheless, the HWE tests showed that the genotype distributions of these SNPs did not deviate from HWE. Furthermore, the age, gender distribution, and BMI of health controls were matched with CHF cases. These data demonstrate that the control group consists of a relatively representative population sample for the present case-control study.

HRH3 is a relatively new histamine receptor subtype that was first identified in central nervous system [7, 21]. This receptor is a Gi protein coupled receptor. Besides its modulating roles in central nervous system, the peripheral functions of HRH3 were also widely explored especially in the cardiovascular system. It was reported that HRH3 was located on the cardiac sympathetic nerve terminals and acted as an important element to modulate the sympathetic neurotransmitter release and hence regulate the cardiac function [9, 22, 23]. More recently, cardiac HRH3 was also found to play variety of other cardioprotective roles such as modulation of cardiac mast cell function [19] and inhibition of local rennin-angiotensin system (RAS) [10] as well as improvement of cardiac hemodynamics and oxidative stress [24]. In addition to the protein functional studies of HRH3, polymorphisms of* HRH3* gene were also found to be related with certain histamine-related diseases such as migraine [25], schizophrenia [26], and breast cancer [16], indicating that variations of* HRH3* gene may have considerable effect on the expression and/or function of HRH3. These lines of evidence strongly agree with the present observation that the* HRH3* rs3787429 polymorphism is associated with CHF risk.

It is noteworthy that, according to the stratified subgroup analyses, the associations of rs3787429 polymorphism with CHF risk were still significant in both ischemic and nonischemic CHF subgroups. These findings further demonstrate that this tag-SNP contributes greatly to the susceptivity of CHF despite the different etiologies. Nevertheless, although the genotype distribution of rs3787429 polymorphism was associated with CHF risk, we did not observe its associations with levels of NYHA class and LVEF, indicating that the* HRH3* locus might just be involved in the initiation of CHF but is unlikely to contribute much in the disease progression.

As for HRH2, previous work also revealed its important role in regulating cardiac function [3, 5, 8]. Furthermore, the rs2067474 polymorphism, located in an enhancer element of* HRH2*, was suggested to induce changes in the expression of receptors [20, 27]. However, we did not observe significant association between this SNP and CHF risk in the present study. As a matter of fact, the significance of association between rs2067474 polymorphism and certain HRH2-related disease varies greatly according to different previous investigations. It was reported that rs2067474 polymorphism was significantly associated with gastric cancer risk [28] but was not associated with risks of developing hypersensitivity to nonsteroidal anti-inflammatory drugs [29] or breast cancer [30]. Therefore, whether and how this SNP affects the expression of HRH2 protein is still unclear and further functional investigation is needed to clarify this issue.

As for gene polymorphisms of the 2 histamine degradation enzymes, we also did not find their associations with CHF risk in the present study. One possible reason is that these variants may not be strong enough to affect susceptivity of CHF although certain SNPs were reported to be connected with lower enzyme activity both in vivo [31, 32] and in vitro [33]. This possibility is reasonable since the two histamine degradation pathways may compensate each other to some extent when one is affected by any genetic variants, which makes the mutations of these 2 degradation enzyme genes less important as compared with those of* HDC* gene [6] in contributing to the induction of CHF.

Several intrinsic limitations of this study should be acknowledged. Although we found the association between* HRH3* rs3787429 polymorphism and CHF risk, the underlying mechanism for this association remains uninvestigated. As rs3787429 is a synonymous tag-SNP that does not cause amino acid change of HRH3, the functional validation of this SNP is relatively difficult. This tag-SNP may either directly affect the function of HRH3 or capture other SNPs that are eventually responsible for the receptor's functional change. Future work regarding the exact underlying mechanisms is greatly warranted. In addition, the studied participants are Han Chinese and the sample size is relatively small. Therefore, replication studies based on CHF patients either from the same ethnic group or other human ethnic groups are still needed for validation of the present findings.

In summary, our present study found for the first time that the rs3787429 polymorphism of* HRH3* gene are significantly associated with the risk of CHF in Chinese Han populations, which may be a novel biomarker for personal prevention and treatment of CHF.

## Figures and Tables

**Table 1:** Primers used for this study.

SNP_ID	Allele	1st-PCRP	2nd-PCRP	UEP_SEQ
*HRH2*				
rs2067474	G/A	ACGTTGGATGACGTGTTTATTACCTGACCC	ACGTTGGATGCTGAGAACCATATCTGGTGC	CATACTCTAAGGCGGTGGC
rs1800689	G/A	ACGTTGGATGCTAAGTGCAAAGTCCAGGTC	ACGTTGGATGATGCACATGATCAGTAGCGG	CCCATCCACCAGCCCGTA
*HRH3*				
rs3787429	C/T	ACGTTGGATGTCACTCAAGAGGGGCTCCAA	ACGTTGGATGTGGGACACCATCTTCATGCG	aATCTTCATGCGCTTCTCCAG
rs3787430	C/T	ACGTTGGATGAGAGGCCGCGCTCACTCAA	ACGTTGGATGACACCATCTTCATGCGCTTC	caaGAGGCCGAGGACGCCGA
*DAO*				
rs2268999	A/T	ACGTTGGATGTTGGGCACGCTGCTTAAACT	ACGTTGGATGAGGAGCTAAGCACTGTTGTC	GCACTGTTGTCATTATTTTCATTTTA
rs10156191	C/T	ACGTTGGATGCTTAGGTCTGAAAACACCCC	ACGTTGGATGGTGGCTGCCATCCTGATGC	CATCCTGATGCTGCAGA
rs1049742	C/T	ACGTTGGATGCGAAGTGCACGTTCAGGAC	ACGTTGGATGAGGGCAACGCTGTGCTCTAC	ccccTCCGGCTGCGCTCCTCCT
rs2071514	G/A	ACGTTGGATGAGACAGTTGAAGTTGTCCGC	ACGTTGGATGTTCGGCGGCACTTTAATTCC	ccgGGCTTCAACTTCTATGC
rs1049748	C/T	ACGTTGGATGCGGCCTTCCGCTTCAAAAG	ACGTTGGATGTTGTGGCCCCAGGGGTTCT	gaCTGGTAAAGAGCAGGTACTT
rs1049793	C/G	ACGTTGGATGGCATCTACCACCAGAACGAC	ACGTTGGATGCAGGGCAGTACCTCATTTTC	ctctaAAAGACCACGGGCGGGT
*HNMT*				
rs11558538	C/T	ACGTTGGATGGCCAAGCAAACTTTACGTTC	ACGTTGGATGTGATGGTGTGTCACCTCTTC	TAGAGCTTGTAGCCAAGA

**Table 2:** Characteristics of CHF patients and control participants.

	CHF (*n* = 333)	Control (*n* = 354)	*P*
Age (years)	62.7 ± 12.3	62.6 ± 8.3	0.909^a^
Male	207 (62.2%)	212 (59.9%)	0.584^b^
BMI (kg/m^2^)	24.3 ± 2.7	23.9 ± 2.8	0.076^a^
Hypertension	202 (60.7%)	93 (26.3%)	**<0.001** ^b^
Dyslipidemia	119 (35.7%)	70 (19.8%)	**<0.001** ^b^
Diabetes	106 (31.8%)	33 (9.3%)	**<0.001** ^b^
Smokers	125 (37.5%)	67 (18.9%)	**<0.001** ^b^
LVEF (%)	33.3 ± 7.0	65.2 ± 6.1	**<0.001** ^a^
Etiology			
Ischemic	180 (54.1%)	—	
Nonischemic	153 (45.9%)	—	
NYHA class			
II	98 (29.4%)	—	
III	130 (39.0%)	—	
IV	105 (31.5%)	—	

BMI: body mass index, LVEF: left ventricular ejection fraction, and NYHA: New York Heart Association.

^a^ *P* values were calculated by Student's *t*-tests.

^b^ *P* values were calculated from two-sided chi-square test.

**Table 3:** Genotype and allele distribution of *HRH2*, *HRH3*, *DAO*, and *HNMT* polymorphisms and their associations with the risk of CHF.

Genotype	CHF	Control	Unadjusted	Adjusted	Adjusted	HWE
	*n* (%)	*n* (%)	*P* ^a^	*P* ^b^	OR (95% CI)^b^	*P* ^c^
*HRH2*						
rs2067474						
GG	240 (72.1)	251 (70.9)	0.477	0.725	0.943 (0.682–1.306)	0.175
GA	86 (25.8)	90 (25.4)				
AA	7 (2.1)	13 (3.7)				
G/A allele	566/100	592/116	0.505	0.720	0.941 (0.676–1.311)	
rs1800689						
GG	311 (93.4)	325 (91.8)	0.341	0.709	0.886 (0.470–1.671)	0.422
GA	21 (6.3)	29 (8.2)				
AA	1 (0.3)	0 (0.0)				
G/A allele	643/23	679/29	0.573	0.709	0.886 (0.468–1.675)	

*HRH3*						
rs3787429						
CC	197 (59.2)	143 (40.4)	**<0.001**	**0.001**	**0.662 (0.523**–**0.838)**	0.067
CT	88 (26.4)	151 (42.7)				
TT	48 (14.4)	60 (16.9)				
C/T allele	482/184	437/271	**<0.001**	**<0.001**	**0.608 (0.470**–**0.786)**	
rs3787430						
CC	229 (68.8)	226 (63.8)	0.371	0.274	0.848 (0.632–1.139)	0.483
CT	89 (26.7)	111 (31.4)				
TT	15 (4.5)	17 (4.8)				
C/T allele	547/119	563/145	0.244	0.258	0.839 (0.619–1.137)	

*DAO*						
rs2268999						
AA	249 (74.8)	263 (74.3)	0.789	0.972	0.994 (0.700–1.411)	0.634
AT	79 (23.7)	83 (23.4)				
TT	5 (1.5)	8 (2.3)				
A/T allele	577/89	609/99	0.754	0.972	0.994 (0.701–1.409)	
rs10156191						
CC	243 (73.0)	269 (76.0)	0.071	0.065	1.391 (0.980–1.975)	0.228
CT	79 (23.7)	82 (23.2)				
TT	11 (3.3)	3 (0.8)				
C/T allele	565/101	620/88	0.158	0.064	1.388 (0.981–1.964)	
rs1049742						
CC	333 (100)	353 (99.7)	1.000	1.000	—	0.979
CT	0 (0.0)	1 (0.3)				
TT	0 (0.0)	0 (0.0)				
C/T allele	666/0	707/1	1.000	1.000	—	
rs2071514						
GG	99 (29.7)	95 (26.8)	0.460	0.511	0.922 (0.724–1.174)	0.932
GA	168 (50.5)	176 (49.7)				
AA	66 (19.8)	83 (23.4)				
G/A allele	366/300	366/342	0.234	0.514	0.923 (0.726–1.174)	
rs1049748						
CC	111 (33.3)	141 (39.8)	0.177	0.420	1.108 (0.864–1.421)	0.686
CT	174 (52.3)	162 (45.8)				
TT	48 (14.4)	51 (14.4)				
C/T allele	396/270	444/264	0.223	0.138	1.204 (0.942–1.540)	
rs1049793						
CC	83 (24.9)	78 (22.0)	0.589	0.513	0.923 (0.727–1.172)	0.739
CG	162 (48.6)	173 (48.9)				
GG	88 (26.4)	103 (29.1)				
C/G allele	328/338	329/379	0.305	0.511	0.923 (0.726–1.173)	

*HNMT*						
rs11558538						
CC	307 (92.2)	320 (90.4)	0.421	0.866	0.951 (0.530–1.707)	0.343
CT	26 (7.8)	34 (9.6)				
TT	0 (0.0)	0 (0.0)				
C/T allele	640/26	674/34	0.431	0.870	0.953 (0.539–1.686)	

HWE: Hardy-Weinberg equilibrium.

Bonferroni's multiple adjustment was applied to the level of significance, which was set at *P* < 0.0045 (0.05/11 SNPs).

^a^ *P* values were calculated from two-sided chi-square tests or Fisher's exact tests.

^b^ *P* and OR (95% CI) values were calculated by logistic regression adjusted for age, gender, body mass index, and traditional cardiovascular risk factors.

^c^HWE *P* values for control group were calculated from two-sided chi-square tests or Fisher's exact tests.

**Table 4:** Multivariate analysis for *HRH3* rs3787429 polymorphism and risk of CHF according to dominant, recessive, and additive genetic models.

SNP	Dominant model	Recessive model	Additive model
	*P* ^a^; OR (95% CI)	*P* ^a^; OR (95% CI)	*P* ^a^; OR (95% CI)
rs3787429	**<0.001; 0.455 (0.322**–**0.642)**	0.450; 0.835 (0.523–1.333)	**0.001; 0.662 (0.523**–**0.838)**

OR: odd ratio; CI: confidence interval.

^a^ *P* and OR (95% CI) values were calculated by logistic regression adjusted for age, gender, body mass index, and traditional cardiovascular risk factors.

**Table 5:** Genotype distribution of *HRH3* rs3787429 polymorphism according to NYHA class and subsets of CHF patients.

	rs3787429			Genotype distribution	Allele frequency
	CC (*n* = 197)	CT (*n* = 88)	TT (*n* = 48)	*P* value	*P* value
NYHA *n* (%)					
II	53 (54.1)	32 (32.7)	13 (13.3)	0.282^a^	0.247^a^
III	74 (56.9)	35 (26.9)	21 (16.2)		
IV	70 (66.7)	21 (20.0)	14 (13.3)		
Hypertension *n* (%)	120 (59.4)	54 (26.7)	28 (13.9)	**<0.001** ^b^	**<0.001** ^b^
Dyslipidemia *n* (%)	66 (55.5)	38 (31.9)	15 (12.6)	**0.017** ^b^	**0.008** ^b^
Diabetes *n* (%)	64 (60.4)	25 (23.6)	17 (16.0)	**0.001** ^b^	**0.005** ^b^
Smokers *n* (%)	70 (56.0)	36 (28.8)	19 (15.2)	**0.008** ^b^	**0.014** ^b^
Ischemic *n* (%)	110 (61.1)	46 (25.6)	24 (13.3)	**<0.001** ^b^	**<0.001** ^b^
Non ischemic *n* (%)	87 (56.9)	42 (27.5)	24 (15.7)	**0.001** ^b^	**0.008** ^b^
LVEF (%)	33.6 ± 6.8	33.4 ± 6.8	31.9 ± 7.9	0.319^c^	—

LVEF: left ventricular ejection fraction; NYHA: New York Heart Association.

^a^ *P* values were calculated from two-sided Fisher's exact tests to analyze the genotype distribution or allele frequency for the *HRH3* rs3787429 polymorphism according to NYHA class.

^b^ *P* values were calculated from two-sided chi-square tests or Fisher's exact tests versus the control population.

^c^ *P* values were calculated by analysis of variance (ANOVA).
